# The Journal: A Swan-Song

**Published:** 1951-10

**Authors:** E. Watson-Williams

**Affiliations:** *Reporter to the* Bristol Medico-Chirurgical Journal 1922; *Editorial Committee* 1926; *Assistant Editor and Editor of Jubilee Index* 1933; *Editor* 1948; *Sometime Honorary Treasurer of the Society of Medical Editors of Great Britain and Ireland*


### THE JOURNAL: A SWAN-SONG

BY

E. WATSON-WILLIAMS, *M.C.*

*Reporter to the* Bristol Medico-Chirurgical Journal 1922;
*Editorial Committee* 1926; *Assistant Editor and*
*Editor of Jubilee Index* 1933; *Editor* 1948; *Sometime Honorary Treasurer of*
*the Society of Medical Editors of Great Britain and Ireland*

---

Our first editor began the tradition that each occupant of the chair should write something about medical journalism. Provincial medical journals have never been numerous in this country; many have perished young. As we shall see, the 1914–18 war caused several old-established journals to suspend publication, although some (not all) resumed about 1926. Even of these, few overcame the mischances of the last ten years. And apparently, if we exclude annual reports of hospitals, transactions of societies and so forth, ours is the only English provincial medical journal to have survived both World wars. In this at least we may feel a modest pride.

The origin of the *Journal* has been well described by L. M. Griffiths (1900: Assistant Editor 1883, PRESIDENT 1887, HONORARY MEMBER), who had himself contributed very largely to it.

" In 1878 it was decided that some of the good work which the Society had done should find a permanent record in a volume of Transactions. Accordingly a volume was issued which, considering the quality of the matter it contained, was published at the exceedingly modest price of five shillings.

Another four years passed, and some members of the Committee thought the time had arrived for the publication of a second volume of Transactions covering that period. Feeling that such a retrospect was at its best by no means satisfactory, and knowing well the ability of my professional brethren in the district, I had the temerity to propose in Committee that a publication with a more frequent periodicity and having the nature of a journal or magazine, should be attempted. This proposition received the support only of Greig Smith and myself. But when the question introduced by Dr. Beddoe was brought before a general meeting of the Society it was determined *nemine contradicente* that the effort should be made, and in 1883 the first number of the *Journal* was issued. It was fortunate in its beginning. In the person of Greig Smith it had an Editor who *con amore* placed at its disposal his commanding and masterly genius.

The second number appeared after an interval of six months, but after the first year it became and has remained a quarterly publication. Its professional success has been great. This is partly testified by the fact that its contributors often find abstracts of their work chronicled in various countries of the world, and partly by its large exchange-list; for, with the exception of some few journals of a special nature which exchange only with their own kind, this includes most of the best medical periodical literature extant. An examination of the exchange-list and of the list of subscribers shows that the *Journal* now travels over the whole globe.

My journal career has shown me that in the work of such a publication there is room for the man of small parts. A successful medical journal must have at the head of its affairs an editor with a well-balanced mind, and among its staff and its contributors those who have clinical and literary accomplishments; but, in addition, it must have someone who will look after its business affairs, see that its matter is methodically arranged and in something like right proportions, and that it goes out with every " i " dotted, and with every " f " unbroken. I part from my work (as Assistant Editor) with many regrets, though it occupied more time than perhaps I was justified in giving to it. I have felt all along that the professional life of a district is the richer for a *Journal*, which worthily gives expression to that life."

Here is J. Greig Smith, our first editor (Editor 1883, PRESIDENT 1893), expressing his opinion on " Modern Medical Journalism " (P.A. 1893) in no uncertain manner:

" There are sundry difficulties which meet the best-intentioned editor which he cannot remove, and which he must accept. One of these, I grieve to say, relates to the deficient literary education of the average medical man. For some mysterious reason the art and science of logic is not expected to be known by medical men. It is not known, and their writings show it. The arts of rhetoric and composition also are ignored; even the humdrum rules of grammar are in abeyance. How much of the editor's function is the humble one of correcting a school exercise in grammar and composition the editor only knows. It is certainly much more than it ought to be, and much more than it need be, if those who guard the portals of medical education were to give some heed to the outcry of those who practise their profession and teach it.

This, then, to begin with. We must accept the fact that, with a few, a very few, and sometimes brilliant exceptions, the literature of medicine at the present day is, as pure literature, either indifferent or positively bad. Scarcely a single issue of a journal is without a paper from which a man educated in ordinary literary methods could not extract abundant examples of errors in logic, in rhetoric, in composition, and even in humble grammar.

Another fact which the editor must accept is the wide-spread ignorance of the history of medicine—recent and remote—which his contributors display. Such ignorance betraying itself on every hand lends colour to the view of certain cynics who hold that it is only by courtesy that our profession is called "learned"; that we are educated by rote, and not by thought; that the evolution of ideas has no interest for us; and that our classics, being dead, may as well be buried. There is ignorance not only of classics, but even of class-books. It would truly seem as if knowledge in our profession began to decay after five years, and was buried and forgotten after ten. However this may be, it is certain that too many writers show a culpable ignorance, or neglect, of the works of others in the fields they seek to cultivate.

The man who earnestly and honestly seeks to keep abreast of real knowledge has to educate himself in a course of training in values whereby he finds out and knows the fool, and the drudge, and the bore and the advertiser; and learns to avoid them. When he has done this, he finds that his labours are not so hard after all. It is, then, a mere turning over of thousands of leaves and a close reading of tens of pages. But it takes a long time, and greatly tries one's patience, to learn whom to avoid in medical literature.

And here our editors might help us. They might select for us. They might silence the chatterer and make the worker talk. The best men and the best workers are silent and retiring; the editor must seek them out, and drag them out, and make them speak. This is not easy matter, as I can from experience testify. It is very hard to get our best men to write."

In the same address Greig Smith spoke of his early elevation to the Presidential Chair as a "reward" for his services as editor. In actual fact this honour has, from our first years, been the traditional due of the *assistant* editor. His post is no sinecure. A mere list of the more important duties would occupy well over a page of this *Journal*: even such a glutton for work as Griffiths had to ask for help.

An Editorial Secretary was accordingly appointed in 1895 to take part of the burden. Curiously, he seems from the first to have been considered more important than the A.E. For example, for the next thirty years *he* presents the annual report. The Editor of course (as now) was appointed by the committee of the Society, not annually but "during pleasure", a provision necessary to secure continuity. But he was not himself a member of that committee, whereas the E.S. seems to have been elected to it each year. The position of the A.E. is obscured by the fact that he was for years Honorary Librarian, and as such *ex officio* member of the committee.

In 1908 new rules provide that the Editor (appointed as before) and the E.S., elected annually, are *ex officio* members of the committee. And each year until 1913 the A.E. seems to have been one of the *elected* members. From then until 1923 no accounts of meetings were published, except for 1919, when the same plan held. In 1923–24 the A.E. was apparently elected as " Past President ". And in 1925 new rules abolished the office of Editorial Secretary. With the indifference of Gallio, the *Journal* continued each year to print the name of the E.S., Dr. A. L. Flemming, who discharged the duties until his death in 1939: the annual report, however, was presented by the Editor or the A.E. Perhaps because in 1925 the E.S. was President, and the A.E. became Editor, no one noticed that the *Journal* had lost half its former representation on the committee. While the office lasted the following held it:

B. M. H. Rogers. Editorial Secretary 1895; PRESIDENT 1910.

J. Taylor. Editorial Secretary 1899; PRESIDENT 1906.

J. A. Nixon. Editorial Secretary 1909; PRESIDENT 1922.

J. M. Fortescue-Brickdale. Editorial Secretary 1913.

A. L. Flemming. Editorial Secretary 1921; PRESIDENT 1926.

R. Shingleton Smith (PRESIDENT 1888, Editor 1892, HONORARY MEMBER), who succeeded, did not write about journalism, perhaps feeling that Griffiths had said all that was required. During his long editorship the *Journal* developed considerably. The work of Lister and the discovery of bacteria had ushered in a medical renaissance. 1883 was the beginning of *The Medical Annual* as well as of our *Journal*. In the early years the bulk of our material consisted of papers and clinical records. But from the very start " Notes on Books " and " Medical Extracts ", a review of recent advances, had found a place.

Under Shingleton Smith the proportions changed. Taking volumes X–XIV, we find only a third is given to original articles. " Progress of the Medical Sciences " and Reviews together occupy more space than this: and in addition there were quite considerable Library Reports, accounts of various Health Resorts, " Scraps ", and reports on " Meetings of Societies " and " Preparations for the Sick ". Under the last head we find not only descriptions of new drugs and appliances, but an occasional hint of some compensations. For example:

" *Preparations for the Sick* "

| Volume | XIII | XIV | XV | XVI |
|---|---|---|---|---|
| Wines . . . | 2 | 3 | 1 | 1 |
| Whisky, etc. . . | – | 1 | 2 | 1 |

The note of restrained wistfulness compels me to quote the last report in full:

" This brand of Scotch Whisky bears on its label the descriptive epithets " real and rare ", " very old ", and " guaranteed pure ". *Its flavour and the absence of after-effects** seem to justify these appellations." Ah, me!

The great increase in the labour of writing abstracts, reviews and reports made necessary the appointment of an Editorial Committee (1891), the function of which has always been " to assist the Editor ". The Editor, of course, remains solely responsible for the conduct and contents of the *Journal*: he need not seek the advice of the Committee, nor take it when sought—there is never any question of a " vote ". Economic pressure led to the abandonment of " Notes on Preparations for the Sick " in 1921 and of " Progress of the Medical Sciences " in 1924. Mainly for similar reasons we have quite recently had to cease publishing " Reviews of Books ". Perhaps, therefore, there will no longer be the former need for an Editorial Committee.

" Meetings of Societies " made its first appearance in Volume IX (1891), with fairly full reports of the discussions at the meetings of the Bristol Medico-Chirurgical Society. Although our origin, from " Transactions ", implies that the bulk of what appears in the *Journal* had already been presented to the Society, it is not possible before this date to discover how much. An addition was made at this stage to the Society's " Rules ", giving

* Our italics.

the Editor the right to publish what was said at meetings. The last issue of Volume X carried a report from the Plymouth Medical Society, and others soon followed.

" Local Medical Notes " began in 1896 (XIV, i) when " Local Representatives " of the *Journal* were appointed in Bath, Cardiff, Exeter, Gloucester, Newport, Plymouth, Swansea and Torquay. In the same volume we find a record of the first use of X-rays in Bristol, and a centenary notice of Jenner with his portrait.

An " Editorial Note " in 1892 (X, 72) records the change in editorship; a second, the appointments just mentioned. After 1900 (XVIII) Editorials became a regular feature.

P. Watson-Williams (Assistant Editor 1900, Editor 1912, PRESIDENT 1913, HONORARY MEMBER), succeeded Shingleton Smith in 1912. His " History of the Bristol Medico-Chirurgical Journal " (1933) appeared so recently that it would be idle to quote from it here. It contains much information about format, printing and the contents of early numbers.

J. A. Nixon (E.S. 1909, A.E. 1912, PRESIDENT 1922, Editor 1926, HONORARY MEMBER), delivered an address on " Provincial Medical Journals " (1932) before the History of Medicine Section of the British Medical Association at the Centenary Meeting. In this he summarised the objects and advantages of a local medical journal which I quoted in our last issue (p. 98). Both the last two editors had to face the difficulties involved in conducting the *Journal* during a major war. These were the subject of comment at the time and have been briefly compared quite recently (1947).

Another change introduced in 1926 was the appointment of the Treasurer of the Society as *ex-officio* business manager of the *Journal.* Whether this is altogether fair is open to question: the two functions are essentially different, and their combination may well deprive one institution or the other of the services of a member who feels them incompatible.

My own very short tenure of office has been remarkable chiefly for a persistent economic struggle, due to a continuous rise in the cost of paper, typesetting, printing and distribution. No sooner did the increase in subscriptions and in advertisement income promise us some relief than a further advance in the expenses of production snatched it away. In an endeavour to demonstrate just where and how " the money goes ", we have regularly published the Balance Sheet of the *Journal*, and with it an " analysis " which brings out clearly that the number of words printed is the determining factor in the cost of production. To reduce this we have eliminated Reviews, Lists of Books Received, Obituary notices and most of the examination results: and can hardly go further in this direction. Too often, alas, we have had to refer to the need for economy, the absolute necessity for brevity, the menace of bankruptcy: for without constant care it would be quite easy to squander a whole year's subscription income on a single quarterly issue—we came within a few pounds of this in 1946. I fear I shall be remembered, if at all, as the Editor who was mad about economy!!

As a more positive attack on our economic difficulties, the appointment of Local Correspondents has been revived: a number of colleagues having kindly undertaken to promote our interests in their areas. At the same time we have tried to devote more space to Local Medical Notes and Proceedings of Societies, and to show that our interests are not confined to Bristol. Many of the senior practitioners in the region were approached and in addition some hundreds of former Bristol students. The mere compilation of the list of the latter was an extremely tedious business and the response not encouraging. Further, in addition to reporting the papers contributed to our own and other societies we have endeavoured to introduce matters of general interest, such as the reports on the Bristol Quadruplets and on " Unusual Children ". A series such as this entails quite a lot of work: we hope it was worth it. At least at the moment the note of moderate optimism in the Editor's Report on page 139 appears to be justified.

We have succeeded also in securing an additional amenity for those subscribers to the *Journal*, whether members of the Society or not, who live at a distance from Bristol. By virtue of a recent agreement they are now entitled to borrow books from the University Library by post.

The other factor that has recently dominated our professional life has been the Health Act. We are all rather conscious of the difficulties inherent in such a fundamental change, particularly on the administrative side: and have been several times reminded of the stringency of materials and of finance which must continue to limit expansion. It is too early to assess how this change will affect medical journalism, although already we can detect an increasing absorption in affairs which tends to make some of our best men reluctant to serve the *Journal*. How interesting now to recall the views expressed by Lord Dawson (1920), Chairman of the Consultative Council, Ministry of Health, when he visited Bristol in 1920 : " A municipalised (Hospital) service was one step forward to the nationalisation of medicine, and that would be a disaster alike to the medical profession and the public."

My own connection with the *Journal* began in 1922 when I was asked to undertake the duties of Reporter of the discussions of our Society: a duty which I continued for some years after joining the Editorial Committee in 1926. The first meeting of that Committee which I attended was number 349 and the last, number 541. After so long a probation it would be scarcely human not to feel disappointment that the tenure of office has been so short. " The labour we delight in physics pain ": but we like the labour to be recognised as well as the delight.

αἰνὸν ἄχος τό μοί ἐστιν, ἐπεὶ πάθον ἄλγεα θυμῶι.
ἀλλὰ τα μεν προτετύχθαι ἐάσομεν. οὐδ' ἄρα πως ῆν
ἀσπερχὲς κεχολῶσθαι ἐνὶ φρεσίν. ΙΛ. Π

### REFERENCES

Griffiths, L. M.—Presentation to, 1900. *Bristol Medico-Chirurgical Journal*, XVIII, 100.

Greig Smith, J.—" Modern Medical Journalism." P.A., 1893. *Ibid.*, XI, 217.

Watson-Williams, P.—" History of *The Bristol Medico-Chirurgical Journal*." 1933. *Ibid.*, L, 151.

Nixon, J. A.—" Provincial Medical Journals." 1932. *Ibid.*, XLIX, 311.

Non-editorial Note, 1947. *Ibid.*, LXIV, 52; cf., p. 85.

Dawson of Penn, Lord.—1920, *B.M.Jl.* 1920, ii, July 17th.

